# Independent review of 4DCT scans used for SABR treatment planning

**DOI:** 10.1002/acm2.12825

**Published:** 2020-02-13

**Authors:** Rachitha Antony, Peta Lonski, Elena Ungureanu, Nicholas Hardcastle, Adam Yeo, Shankar Siva, Tomas Kron

**Affiliations:** ^1^ Department of Physical Sciences Peter MacCallum Cancer Centre Melbourne Vic Australia; ^2^ Centre for Medical Radiation Physics University of Wollongong Wollongong NSW Australia; ^3^ Department of Radiation Oncology Peter MacCallum Cancer Centre Melbourne Vic Australia; ^4^ Sir Peter MacCallum Department of Oncology University of Melbourne Melbourne Vic Australia

**Keywords:** 4DCT, motion management, radiotherapy, SABR

## Abstract

Four‐dimensional computerized tomography (4DCT) is required for stereotactic ablative body radiotherapy (SABR) of mobile targets to account for tumor motion during treatment planning and delivery. In this study, we report on the impact of an image review quality assurance process performed prior to treatment planning by medical physicists for 4DCT scans used for SABR treatment. Reviews were performed of 211 4DCT scans (193 patients) over a 3‐yr period (October 2014 to October 2017). Treatment sites included lung (n = 168), kidney/adrenal/adrenal gland (n = 12), rib (n = 4), mediastinum (n = 10), liver (n = 2), T‐spine (n = 1), and other abdominal sites (n = 14). It was found that in 23% (n = 49) of cases patient management was altered due to the review process. The most frequent intervention involved patient‐specific contouring advice (n = 35 cases, 17%) including adjustment of internal target volume (ITV) margins. In 13 cases (6%) a rescan was requested due to extensive motion artifact rendering the scan inadequate for SABR treatment planning. 4DCT review by medical physicists was found to be an effective method to improve plan quality for SABR.

## 1 INTRODUCTION

Stereotactic ablative body radiotherapy (SABR) is characterized by high radiation doses delivered in one or few treatment fractions. SABR has been shown to be safe and effective for patients with early‐stage non‐small cell lung cancer^1^, ^2^, ^3^ and kidney cancer,^4^, ^5^ and shown promise for liver,^6^, ^7^ spine^8^ and oligometastatic disease,^9^, ^10^ as well as pancreas and prostate,^11^ in select patients. SABR treatment requires image guidance for accurate delivery, particularly for mobile targets. Patient immobilization and motion management strategies are used to ensure treatment is delivered as planned. For mobile targets, retrospectively binned 4D‐computed tomography (4DCT) scans may be performed to generate volumetric images at each phase of the breathing cycle. From the tumor motion in the individual phases, one can generate an internal target volume (ITV) which encompasses the GTV as well as its motion. Due to the risk of artifacts in 4DCTs, our institution has adopted a policy that these scans are reviewed by a medical physicist prior to treatment planning to ensure that the image is suitable for approximation of the tumor motion due to respiration as well as for the creation of a reasonable reference image for image guidance. Ideally, the ITV contour must encompass the size of the tumor as well as its full excursion throughout the entire respiratory cycle.

Irregular breathing patterns or 4DCT reconstruction errors such as one phase not reconstructing properly may lead to systematic errors in ITV delineation propagating through the treatment chain which may not be obvious upon reviewing the maximum intensity projection (MIP) or average scans alone. Recently the European Organisation for Research and Treatment of Cancer (EORTC) multicentre Lungtech trial reported on the results of RTQA activities for 4DCT across 11 centers. Large deviations in contour volume of up to 99% were found across different sites despite imaging the same phantom under the same motion pattern.^12^ The effects of irregular breathing patterns on ITV delineation of moving targets in the context of lung SABR have been described by Clements et al.^13^ who demonstrated erroneous ITV delineation using MIP images for moving targets with large amplitude undergoing irregular motion patterns. Similar findings have been reported by Park et al.^14^ who determined that the MIP underestimated the true target motion in the case of irregular motion. Measured PTV dose discrepancies of greater than 10% were reported by Huang et al.^15^ for irregular motion patterns in a moving phantom for targets with large excursions, demonstrating systematic under‐dose of the PTV periphery in such cases. Clinical consequences may be severe, since systematic PTV under‐dosing from inappropriate ITV delineation will result in compromised tumor control probability. This risk is heightened in the superior‐inferior direction for co‐planar field deliveries where the dose falloff is steepest. Therefore, it is essential that the appropriateness of all 4DCT imaging be verified prior to clinical use to ensure that images derived are a true representation of the full tumor excursion, particularly in cases of irregular breathing.

This study presents the findings of independent, prospective reviews performed by radiation oncology medical physicists of 211 patient 4DCT scans acquired for SABR pretreatment planning in a large radiotherapy centre. We report on the frequency of required intervention as a result of the review process and correlation with regularity of patient breathing trace.

## 2 MATERIALS AND METHODS

Review guidelines for 4DCT image sets were developed based on commissioning work^16^ and experiences from quality assurance for several clinical trials.^17^, ^18^ An in‐house training programme was developed for medical physicists to establish a minimum skillset for performing 4DCT reviews in the context of SABR. A patient‐specific review checklist was designed to aid in the review process and facilitate data collection, which has been provided as supplementary material.

4D‐computed tomography scans were acquired on a Brilliance widebore 16‐slice scanner (Philips Medical Systems, Eindhoven, the Netherlands) using retrospective gating with a gantry rotation period of 0.44 s, 140 kVp and a pitch adjusted based on the breathing rate with a resulting patient dose approximately twice the one of a 3D scan.^19^ 4DCT was also performed for lesions where dose calculation was likely to be affected by surrounding mobile structures, such as ribs and lower thoracic spine at the level of the diaphragm. Respiration was monitored using the Philips bellows system affixed to the patients’ abdomen.^20^ Audio or visual coaching was not routinely used however if irregular breathing was noted during the surview scan, radiation therapists would pause the scan procedure to provide basic coaching, although some patients still could not breath regularly throughout the entire scan. The resulting respiratory trace was used for phase binning, creating 10 phases of the breathing cycle. Maximum intensity projection (MIP) and average datasets, which are used for ITV delineation and dose calculation, respectively, were reconstructed from the raw data. The MIP was used for ITV delineation. 4DCT scans were reviewed by a medical physicist prior to treatment planning. Review was performed on the CT console, using the PulmoViewer application. This application allows visualization of the breathing trace with the 4DCT image data, as well as tools to determine the corresponding breath at each superior‐inferior scan location. Tumor motion was measured using the ruler tool provided in PulmoViewer to assess the maximum displacement of the lesion between maximum inhale and exhale phases. The tumor boundaries were identified using the radiation oncologists contour when available, or through diagnostic imaging in consultation with radiation oncologists. A single, well‐defined edge of the tumor on each phase was used to determine motion, therefore this is an estimate of tumor motion rather than the motion of the centre of mass. Choice of tumor edge was at the discretion of the reviewing physicist and was case‐specific, though usually the inferior‐most aspect of the lesion was chosen if well‐defined. If the 4DCT was deemed by the medical physicist to not be an accurate representation of tumor motion, advice was provided on whether to rescan the patient or adjust planning target volume (PTV) margins to account for increased uncertainty, along with an estimate of the uncertainty.

**Figure 1:**
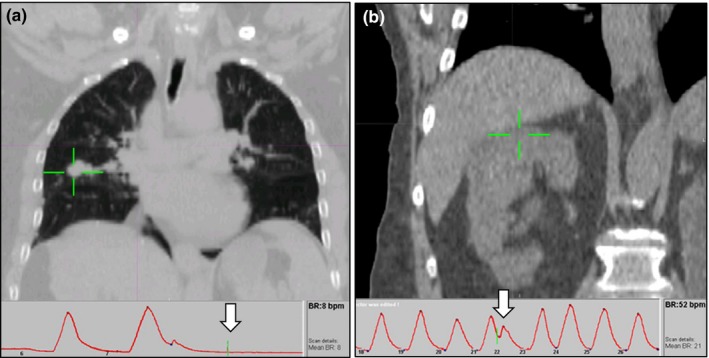
Examples showing irregular breathing in the case of (a) breathing stopped during scanning at the tumor level, and (b) irregular breath at the tumor level despite otherwise regular breathing. The cross‐hairs indicate that the tumor and the arrows mark the breathing track at the tumor level

4D‐computed tomography review data were collected from three radiotherapy facilities across our institution over a 3‐yr period between October 2014 and October 2017. Outcomes of the 4DCT reviews were assessed and each patient breathing trace were classified according to regularity. Respiration cycles were classified as either “regular”, “adequate”, or “irregular”. For a breathing trace to be considered “regular”, the breathing pattern had to be consistent, repetitive in its amplitude and frequency, and free of significant irregularities, such as a halt in breathing or considerable change in breathing pattern. “Adequate” scans contained some irregularities, such as a change in breathing pattern, but not affecting the tumor level. “Irregular” scans contained considerable irregularity in breathing pattern at some point during scanning level of tumor excursion, or a change in breathing at the tumor level severe enough such that the subsequent image would not fully capture the tumor motion. Examples of “irregular” breathing traces at the tumor level are shown in Fig. 1. Breathing classification was made qualitatively, based on the judgment of the reviewing medical physicist. Additionally, the tumor size and motion was documented for each case, including whether hysteresis was evident in the tumor excursion throughout the respiratory cycle. Hysteresis was determined by observation of tumor motion on all phases viewed on the sagittal plane. Tumor motion in the anterior‐posterior direction as well as superior‐inferior was classified as containing hysteresis. Reported CT dose index (CTDI), pitch and breathing rates were also recorded. Breathing rates were measured using the tool provided in the PulmoView software, which reports both the average and location‐specific breathing rate as chosen by the user. The outcomes from each review regarding patient management were also assessed.

## 3 RESULTS

Between October 2014 and October 2017, a total of 597 patients scanned with 4DCT were treated using SABR. Of those, 211 4DCT scan records were available for this retrospective audit. Target locations included lung (n = 168), kidney/adrenal/adrenal gland (n = 12), rib (n = 4), mediastinum (n = 10), liver (n = 2), T‐spine (n = 1) and other abdominal sites (n = 14). Review of 4DCT scans required approximately 20 min of medical physicists’ time per patient. As the SABR programme increased capacity, the number of 4DCT reviews was found to steadily increase.

**Figure 2:**
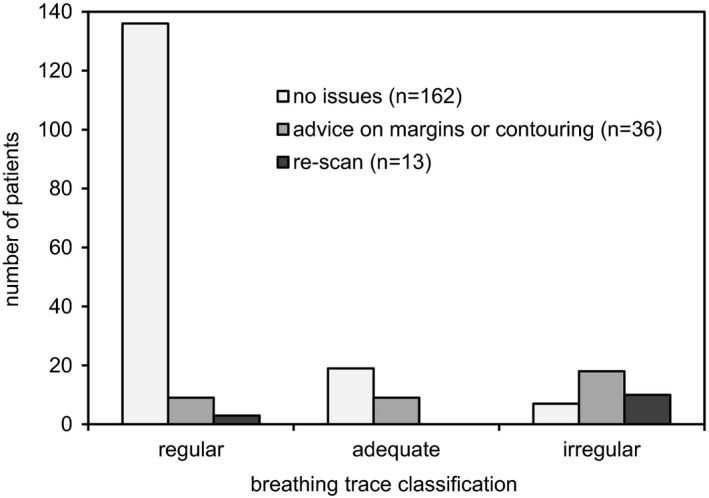
Distribution of patient breathing traces according to respiratory cycle regularity for 211 SABR patients. Change in patient management as a result of 4DCT review is indicated by the shaded bars. A total of 49 cases (23%) required change in patient management. Of those, 25 (51%) were classified as ‘adequate’ or ‘irregular’ breathing

The number of patient breathing traces which were considered “regular”, “adequate” or “irregular” is shown in Fig. 2. The impact on patient management for each category is also shown. No issues were found for 162 patients (77%) and the scans were used for SABR treatment planning without intervention. Of those 162 patients, 136 (84%) had regular breathing patterns, 19 (12%) had adequate regularity and 7 (4%) were considered irregular. For remaining cases (n = 49, 23%), 4DCT reviews revealed issues with the final images and required intervention. A re‐scan was subsequently requested in 13 cases (6%) due to excessive motion artifact rendering the final images unsuitable for ITV delineation for SABR treatment planning. For remaining cases (n = 35, 17%), advice to use modified margins in ITV delineation or other contouring advice including fusion of staging images such as PET was provided to compensate for deficiencies in the 4DCT scan based on advice from the reviewing medical physicist.

**Figure 3:**
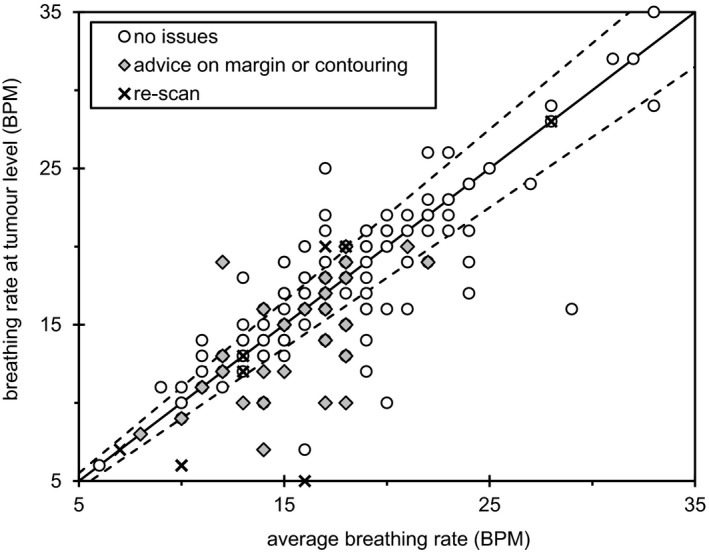
Correlation between average breathing rate throughout the 4DCT scan duration and breathing rate at the tumor level. The solid line represents the line of identity and the dashed lines represent ± 10% variation

Figure 3 shows the average breathing rate throughout the 4DCT scan and recorded breathing rate at the tumor level, with data grouped according to intervention type. The line of identity is shown by a solid line with a ±10% margin indicated by the dashed lines. It can be seen that breathing rate at the tumor level compared to breathing rate throughout the scan did not necessarily predict intervention requirements.

**Figure 4:**
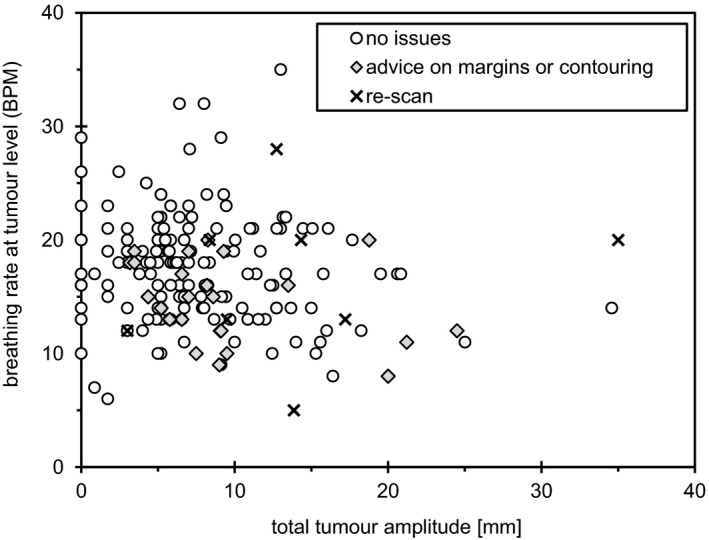
Change in patient management is shown relative to tumor amplitude and patient breathing rate (breaths per minute, BPM) at the tumor level. Motion less than 3 mm required no intervention. Breathing rate was not a predictor for intervention requirements

The amplitude of total tumor motion is shown in Fig. 4 as a function of breathing rate at the tumor level, with data grouped according to intervention requirements. Tumors with motion less than 3 mm did not require intervention regardless of breathing rate. Large tumor excursion or rapid breathing rate were not predictors for intervention.

Table 1 shows the frequency of tumor hysteresis throughout the respiratory cycle. Hysteresis was observed in 30% of patients in this study and is often noted for inferiorly located lesions close to the posterior chest wall.

**Table 1:** Summary cases involving tumor hysteresis. Hysteresis was observed in 64 out of 211 4DCT scans (30%). For the 48 cases requiring some change in patient management, 23 cases (48%) were observed to have tumor hysteresis compared with 41 out of 163 (25%) of cases where no intervention was required

Hysteresis	No issues		Re‐scan or advice	
	n	%	n	%
Yes/Slight	41	25	23	48
No	122	75	25	52

Comments in the review form were reviewed to determine the cause of the artifacts. A number of common causes were identified:

1. The patient’s breathing was highly irregular, leading to poor tumor definition in any one phase, and insufficient quality to determine range of tumor motion.
2. The patient was breathing regularly, but coughed during the acquisition
3. Patient was breathing regularly, but while the tumor was moving through the scanning plane the patient stopped breathing, leading to the tumor appearing artificially stationary, with anatomy superior and inferior moving with respiration.
4. Patient’s breathing continuously slowed down from initial scan pitch setting to acquisition. This may have been due to medication to relax the patient for the scan
5. The patient did not exhale (or inhale) fully, while scanning through the superior (or inferior) aspect of the tumor. This resulted in the superior (or inferior) aspect of the tumor at full expiration (inspiration) not being recorded, i.e., lack of information on either end of the tumor excursion.
6. The patient had an unintended deep inspiration while the tumor was moving through the scanning plane, leading to overestimation of tumor motion

Reasons for physics consultations other than due to breathing irregularity and motion estimation included overestimation of the required tube current by the scanner software, slow breathing patterns (<10 bpm not allowing 4DCT acquisition), inaccurate detection of inhale peaks by the scanner software and poor image quality.

## 4 DISCUSSION

This study reports on the outcomes of independent review for patient 4DCT scans acquired for treatment of SABR to mobile targets. The aim of these reviews was to determine if each scan was a reasonable representation of tumor motion throughout the breathing cycle and was appropriate for the purposes of SABR treatment planning, including target (ITV) delineation and dose calculation.

One limitation of this study is the subjectiveness amongst different physicists in performing quantitative analysis of patient 4DCT reviews. While training was provided to harmonize interpretation, there is still a degree of subjectiveness in the review process. Nevertheless, intervention was required in 23% of all reviewed cases. Irregular breathing rate was found to be a contributor to inadequate scans (16% of regular breathing traces requiring intervention compared to 57% of scans classified as “irregular”, Fig. 2). One common problem was identified as inappropriate choice of scan pitch. Scanner pitch is adjusted based on patient breathing rate prior to commencing a scan. A lower pitch is required to maximize the chance of fully capturing tumor motion in the case of slower breathing rates. The pitch is selected after the patient has spent some time in quiet breathing and is monitored up until commencing a scan. However, upon commencing a scan it was found in some cases that a patient breathing rate can change, even throughout the duration of the scan. In some cases the breathing stopped completely while scanning through the level of the lesion, resulting in no visible tumor motion. In such cases a rescan is required which usually addressed concerns raised in the first scan, unless a similar interruption in breathing pattern occurred. In some cases irregular breathing was noted during the scan but no intervention was required. This may be due to the irregularity occurring at anatomical locations away from the target region. In such cases, irregular breathing is noted but if the target region is unaffected intervention is not warranted. Since changes in breathing rate were shown to be a significant contributor to motion artifacts in our centre, radiation therapists have subsequently begun monitoring the respiratory trace closely during a scan. If irregular breathing is indicated during a scan, a physicist is called to review the respiratory trace while the patient is still on‐site. This facilitates more timely re‐scans where warranted without the need to call a patient back to hospital.

Figure 1 shows that both large [Fig. 1(a)] and quite subtle [Fig. 1(b)] irregularities can impact on motion assessment. Both breathing frequency and amplitude can have a detrimental impact. Through the examples shown in this study, amplitude can have a major impact if the tumor isn’t moving its “normal” extent during acquisition then tumor motion will not be sufficiently captured. However, irregularities in frequency also impact our assessment due to discontinuity artifacts, which is often inter‐related to image acquisition parameters such as pitch factor and gantry speed. It is thus quite challenging to quantify respiratory trace irregularities in a manner that can be applied routinely in the clinic. Thus, ongoing patient‐specific reviews are required.

Typically a 4DCT scan acquires images of each anatomical slice for the duration of one to two breaths. Just one irregular breath can therefore distort the resulting image at a given anatomical slice. Review of PET scans (if available) acquired over several minutes was used to augment the relevant information where necessary. Also the CBCT, or 4D‐CBCT if available, on the first treatment day can be used to validate the motion estimates. 4D cone‐beam CTs were occasionally acquired to evaluate motion, as these are more robust to breathing irregularity due to the whole anatomy being imaged for at least 2 min worth of breathing. It should be noted that due to the fact that 4DCTs are only acquiring motion from 1 to 2 breaths, coupled with the sampling frequency, the treatment respiratory motion is underestimated in 4DCTs.^21^ This means that any underestimation of the motion from 4DCTs is potentially more significant relative to treatment motion.

Tumor hysteresis was noted in 30% of cases (n = 64). Of those, 48% required intervention compared to 25% of cases without hysteresis. Although this study is not powered to compare intervention rates with and without tumor hysteresis the differences are worth noting. It may be that a more complex motion pattern has a higher chance of being missed in the presence of artifacts, compared to a more simple superior/inferior motion pattern.

Earlier studies suggest that artifacts in 4DCT are common and associated with breathing irregularity.^22^ Patient training, coaching and feedback would be helpful to improve patient compliance with regular and reproducible breathing.^23^ Furthermore, thoracic lesions are subject to often complex motion patterns depending on the location and can even be affected by cardiac motion.^24^ Individualized ITVs based on respiratory‐gated 4DCT are therefore necessary for improving target definition.^25^ The additional anterior‐posterior and left‐right motion requires careful consideration of each phase of the breathing cycle, since the maximum inhale and maximum exhale may not capture the intermediate motion patterns. Use of the maximum intensity projection (MIP) image or all individual phases for ITV delineation ensures tumors with hysteresis are fully captured.

## 5 CONCLUSIONS

Patient‐specific 4DCT reviews by a medical physicist was shown to have a significant impact on patient management in a large cohort of patients treated with SABR to moving lesions with a high intervention rate of 23% of all cases. Irregular breathing patterns during 4DCT scans were shown to cause artefacts which may impact on the resulting ITV contours, hence treatment fields. In 23% of cases the physicist was able to advise on margins to accommodate for lost motion during the scan, while in other cases a rescan was required. Tumor hysteresis was noted in 30% of scans, requiring careful review of all phases to ensure tumor excursion is fully captured in all directions of motion. Results from this study suggest patient‐specific 4DCT QA should be a mandatory part of a patient’s treatment pathway in SABR treatments of moving targets to ensure motion is adequately captured for the purposes of motion management and treatment planning.

## CONFLICT OF INTERESTS

The author have no relevant conflict of interest to disclose.

## Supporting information

Fig. S1 Physics ticksheet used to record results of Physicist reviews of 4DCT scans used for SABR treatment planning.
