# Supplementary material for: Independent review of 4DCT scans used for SABR treatment planning
Source: J Appl Clin Med Phys. 2020 Feb 13;21(3):62–7. doi: 10.1002/acm2.12825 (PMC7075381; doi:10.1002/acm2.12825)
Supplement: Supplementary file 1 — Fig. S1 Physics ticksheet used to record results of Physicist reviews of 4DCT scans used for SABR treatment planning. [file ACM2-21-62-s001.docx]

**Supplementary Material:**

Figure S1 – Physics ticksheet used to record results of Physicist reviews of 4DCT scans used for SABR treatment planning.

|  | **Physics Check Sheet for 4DCT** | | | | | |  |  |  |
| --- | --- | --- | --- | --- | --- | --- | --- | --- | --- |
|  |  |  |  |  |  |  |  |  |  |
|  | Patient Name, UR: | | | |  |  |  |  |  |
|  |  |  |  |  |  |  |  |  |  |
|  | Expected treatment start date: | |  | |  |  |  |  |  |
|  | Date of 4DCT: | |  | |  |  |  |  |  |
|  | Reviewing physicist 4DCT/PLAN | |  |  |  |  |  |  |  |
|  | Clinical trial | |  | |  |  |  |  |  |
|  | **4DCT Check Date:** | | **Check/Comments** | |  |  |  |  |  |
|  | Treatment site | |  | |  |  |  |  |  |
|  | Lesion location | |  | |  |  |  |  |  |
|  | Breathing type (J…) | |  | |  |  |  |  |  |
|  | Phase for exhale | |  | |  |  |  |  |  |
|  | Breathing rate at SI level of tumour | |  | |  |  |  |  |  |
|  | Average/range breathing rate | |  | |  |  |  |  |  |
|  | Regular breathing at SI level of tumour | |  | |  |  |  |  |  |
|  | Tumour amplitude (mm, SI:LR:AP) | |  | |  |  |  |  |  |
|  | No artefacts affecting tumour | |  | |  |  |  |  |  |
|  | Approximate lesion diameter | |  | |  |  |  |  |  |
|  | Hysteresis? | |  | |  |  |  |  |  |
|  | Suitable for gating? | |  | |  |  |  |  |  |
|  | AVG appropriate | |  | |  |  |  |  |  |
|  | MIP appropriate | |  | |  |  |  |  |  |
|  | CTDI/DLP/mAs | |  | |  |  |  |  |  |
|  | Pitch | |  | |  |  |  |  |  |
|  | **4DCT Comments** | |  | |  |  |  |  |  |
|  |  | | | |  |  |  |  |  |
|  |  | | | |  |  |  |  |  |
|  | **Signature:** | **Date:** | | |  |  | |  |  |
|  | **General Comments** | | | |  | | | | PDF issued to RT; Mosaiq reporting complete; SABR QA spreadsheet |
|  |  |  |  |  |  |  |  |  |  |
|  |  |  |  |  |  |  |  |  |  |
